# Identifying Cytochrome P450 Functional Networks and Their Allosteric Regulatory Elements

**DOI:** 10.1371/journal.pone.0081980

**Published:** 2013-12-03

**Authors:** Jin Liu, Gregory J. Tawa, Anders Wallqvist

**Affiliations:** Department of Defense Biotechnology High Performance Computing Software Applications Institute, Telemedicine and Advanced Technology Research Center, United States Army Medical Research and Materiel Command, Fort Detrick, Maryland, United States of America; University of Cantebury, New Zealand

## Abstract

Cytochrome P450 (CYP) enzymes play key roles in drug metabolism and adverse drug-drug interactions. Despite tremendous efforts in the past decades, essential questions regarding the function and activity of CYPs remain unanswered. Here, we used a combination of sequence-based co-evolutionary analysis and structure-based anisotropic thermal diffusion (ATD) molecular dynamics simulations to detect allosteric networks of amino acid residues and characterize their biological and molecular functions. We investigated four CYP subfamilies (CYP1A, CYP2D, CYP2C, and CYP3A) that are involved in 90% of all metabolic drug transformations and identified four amino acid interaction networks associated with specific CYP functionalities, i.e., membrane binding, heme binding, catalytic activity, and dimerization. Interestingly, we did not detect any co-evolved substrate-binding network, suggesting that substrate recognition is specific for each subfamily. Analysis of the membrane binding networks revealed that different CYP proteins adopt different membrane-bound orientations, consistent with the differing substrate preference for each isoform. The catalytic networks were associated with conservation of catalytic function among CYP isoforms, whereas the dimerization network was specific to different CYP isoforms. We further applied low-temperature ATD simulations to verify proposed allosteric sites associated with the heme-binding network and their role in regulating metabolic fate. Our approach allowed for a broad characterization of CYP properties, such as membrane interactions, catalytic mechanisms, dimerization, and linking these to groups of residues that can serve as allosteric regulators. The presented combined co-evolutionary analysis and ATD simulation approach is also generally applicable to other biological systems where allostery plays a role.

## Introduction

Cytochrome P450 (CYP) enzymes provide important defense mechanisms against harmful endogenous and exogenous chemicals [1]. Human CYPs can be categorized into 18 families and 44 subfamilies, with 8 subfamilies involved in xenobiotic metabolism, namely CYP1A, CYP2A, CYP2B, CYP2C, CYP2D, CYP2E, CYP2F, and CYP3A. Among these, and in order of importance, CYP3A, CYP2C, CYP2D, and CYP1A are responsible for metabolizing >90% of all drugs. Furthermore, these enzymes are responsible for most drug-drug interactions where the presence of one drug alters the metabolism of another drug, potentially altering the pharmacological properties of either drug and leading to adverse toxic effects. Despite extensive efforts in past decades to understand the mechanism behind these effects, drug metabolism and drug-drug interactions remain difficult to predict, partially due to the observed complex allosteric regulation and cooperative behaviors [2-5]. For example, CYP3A4, one of the most important isoforms that is responsible for >50% of all drug metabolism, exhibits atypical kinetics due to allosteric effects [6]. Recently, Woods et al. reported that the allosteric effector α-naphthoflavone (ANF) changes the product ratio in the sequential metabolism of Nile red (NR), a CYP3A4 substrate [7]. However, the specific allosteric mechanism for this system remains unknown. To begin to understand the mechanism behind these observations, we needed to create a systematic approach that allows us to identify putative allosteric sites. Here, we present combined co-evolutionary analysis and molecular dynamics simulations to understand the allosteric regulation of CYP metabolism.

For individual proteins, evolutionary pressures tend to conserve key residues in catalytic sites and those that maintain protein-protein interactions [8]. Importantly, residues far away from the active site not involved in protein-protein interactions can also be co-evolved above the evolutionary noise level. These co-evolved residues are typically involved in poorly understood long-range interactions with key functional residues [9] that are as important for the biological function of the protein as active site residues [10]. We can quantitatively measure the co-evolution of residues in aligned sequences and identify clusters of residues that follow similar patterns of co-evolution. Theoretically, we could use these patterns to generate intra-protein networks by identifying all residues co-evolved with residues known to be functionally important. 

Several methods investigating co-evolutionary networks in proteins have been proposed, including sequence-based statistical methods [11], statistical approaches coupled with phylogenetic information [12], and non-equilibrium molecular dynamics simulation methods [13]. Here, we used the sequence-based Maximal SubTree (MST) combinatorial method to detect the functionally important co-evolved residue networks of different CYP isoform families using phylogenetic information [14]. This method successfully detected mechanical and functional networks for hemoglobin and serine protease families [14].

The group of residues involved in the communication relevant for one aspect of protein function constitutes a function-specific network. We successfully identified such functional networks for each of the main four CYP isoforms subfamilies involved in drug metabolism. These networks were associated with membrane binding, heme binding, catalytic activity, and enzyme dimerization. We further examined the allosteric nature and regulation of reaction products via the heme-binding network in CYP3A4 using anisotropic thermal diffusion (ATD) [13] molecular dynamics simulations. ATD detects the presence of long-range residue-residue interactions and has been used to predict allosteric sites and to identify signaling pathways in postsynaptic density protein 95 (PSD-95) [13]. The fundamental idea behind ATD is to locally heat selected groups of one or more spatially close residues or co-factors while keeping the rest of system in a super-cooled state (temperature: 10 K). This procedure suppresses thermal fluctuations that could otherwise obscure the small-amplitude correlated motion associated with long-range allosteric interactions. This reduces the overall thermal motion for the system (noise), whereas correlated motion across the allosteric network is enhanced (signal). In this way, the signal-to-noise ratio increases, thereby facilitating the identification of allosteric sites. We showed that our prediction of residues F419 and W407 from a co-evolutionary analysis of CYP3A4 was consistent with an allosteric regulation mechanism of this enzyme. In particular, we found that F419A and W407A mutants allosterically change the dynamic behavior of the residues at the substrate binding sites, which may subsequently change the metabolic products of the substrate. The combined co-evolutionary analysis and ATD simulation approach described here is of general utility and may be used in other biological systems to identify allosteric sites.

## Results

### Co-evolutionary analysis identified four functionally distinct CYP networks

**Figure 1:**
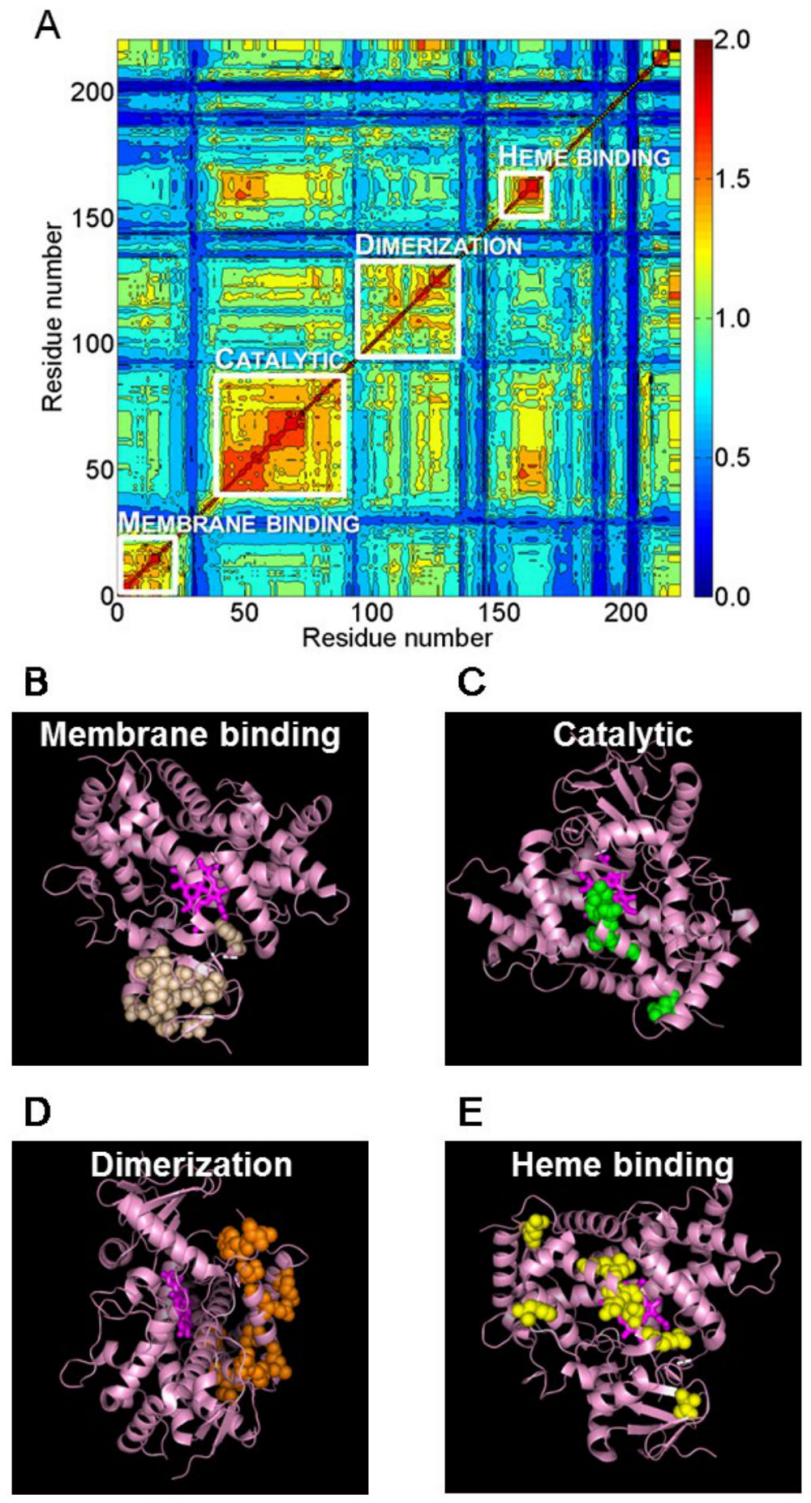
Networks detected by co-evolutionary analysis of CYP2C sequences. *A*: heat map showing the co-evolutionary score for each pair of residues tagged as evolving together above the evolutionary noise. Not all residues in the CYP2C sequences are shown, and the order of the residues was determined via clustering based on the co-evolutionary score. The residue clustering identified four primary groupings that we characterized as a membrane-binding network, a catalytic network, a dimerization network, and a heme-binding network. Although the networks are separable by their primary function, cross-talk can occur between these networks as is evident from the off-diagonal elements. Each pairwise entry was color coded from high (red) to low (blue) co-evolutionary scores. Each residue network was further mapped to the atomic structure of CYP2C9 (PDB code 1R9O) in *B*-E and shown in the following different orientations: the membrane-binding network (*B*), the catalytic network (*C*), the dimerization network (*D*), and the heme-binding network (*E*). The heme molecule is shown in a stick representation, and the residues belonging to the functional networks are shown as spheres.

We performed co-evolutionary analysis for the four most important drug metabolizing CYP1A, CYP2C, CYP2D, and CYP3A subfamilies. Using the CYP450 Engineering Database [15], we downloaded 199, 157, 79, and 191 sequences for the CYP1A, CYP2C, CYP2D, and CYP3A subfamilies, respectively. The pair-wise sequence identities are 13-100% for each subfamily. We performed multiple sequence alignments for each sequence family and calculated the corresponding distance trees. We selected, evaluated, and clustered co-evolved sequence positions to form groups or networks of co-evolved residues [14]. We then mapped these networks to the corresponding molecular structures of each of the different CYP isoforms and identified the putative biological process or function that could be associated with each network. Figure 1A shows the all-to-all pair-wise relative co-evolution scores of the selected co-evolved residues for the CYP2C subfamily. Groups of co-evolved residues were apparent by inspection and we selected four clusters for further analysis. After mapping the selected residues in each network to the CYP2C9 structure, we identified the functional significance of each network based on literature evidence [16-20]. In this way, we classified the four networks shown in Figure 1A as involved in membrane binding, catalytic function, dimerization, and heme binding. Figures 1B-E show the location of these residue networks on a molecular model of CYP2C9. The corresponding results for the CYP1A, CYP2D, and CYP3A families are shown in Figure S1. Interestingly, we could not identify networks associated with substrate binding, implying that substrate recognition is specific to each species of the subfamily as the substrate recognition residues are not co-evolved in the subfamily. 

#### Membrane-binding networks

**Figure 2:**
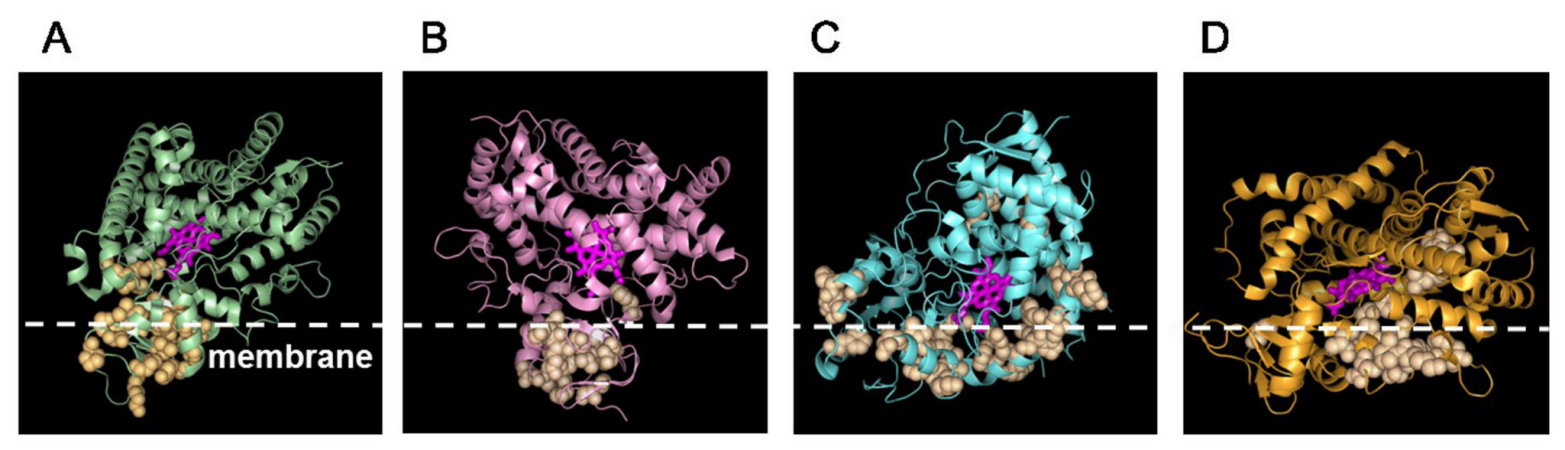
Membrane-binding networks for different CYP isoforms. Residues identified as belonging to membrane-binding networks were mapped to the molecular structures of CYP1A (PDB code 2HI4; *A*), CYP2C (PDB code 1R9O; *B*), CYP3A (PDB code 1W0E; *C*), and CYP2D (PDB code 2F9Q; *D*). The location of the membrane-bound portion of the enzyme partly determines which substrates can access each enzyme. The heme molecule is shown in a stick representation, and the residues belonging to the functional networks are shown as spheres. The position of the membrane line was qualitatively positioned based on the location of the residues in the membrane-binding networks.

One of the recurring networks detected for all the isoforms was the membrane-binding network. We based this network designation primarily on residue proximity to the N-terminal trans-membrane helix, which is normally imbedded in the cell membrane [16,19]. However, the number and position of residues involved in this network were different for the various CYP isoforms. For the CYP1A subfamily, one of the networks we detected included 26 residues distributed between positions 40 and 111 in the sequence, including residues that connect directly to the N-terminal trans-membrane helix. Similarly, for the CYP2C family, we found 18 residues located between positions 29 and 88 in the sequence as a membrane-binding network. The CYP2D and CYP3A subfamilies included more residues in their membrane-binding network (62 and 45 residues, respectively) and encompassed both residues that were strongly associated with membrane binding sites as well as residues that were far away from these sites, as judged by the enzyme structure. Figure 2 shows where the mapped membrane binding networks were located on the different CYP isoform structures and plausible locations of the membrane interface based on the position of the residues involved in the networks. We noticed that each of the CYP1A2, CYP2C9, CYP2D6, and CYP3A4 isoforms adopts different orientations with respect to the membrane, suggesting a functional role of the enzyme/membrane interaction. Under physiological condition, CYPs are partially exposed to the aqueous phase of the cytosol and partially embedded in the cell membrane. This allows CYPs to potentially access and process substrates from both the aqueous phase and the membrane bound fraction. We hypothesized that the orientation CYP isoforms adopt at the membrane interface is critical in determining/selecting active-site access paths of the substrate. To characterize these different membrane orientations, we analyzed the accessible paths to the interior molecular voids for the subfamilies (see METHODS for details and Figure S3). The opening of the access channel for CYP2C was embedded in the membrane, whereas for CYP1A the opening of the access channel was toward the aqueous solvent phase. Similarly for CYP2D and CYP3A, the substrate access channel faced the solvent, consistent with the hydrophilic nature of the compounds processed by these enzymes [20].

To validate our prediction, we searched the literature and found two experimental models for the CYP2 family [21,22]. Our predicted model of CYP2C9 was in good agreement with the experimental models. The comparison of our predicted model and the experimental model is shown in Figure S2A. We further performed 25 ns MD simulation using an implicit membrane model [23] for CYP2C9. The simulation showed no significant conformational re-orientation of the CYP2C9 in this membrane model. The RMSDs of the simulation are shown in Figure S2C. 

#### Catalytic networks

**Figure 3:**
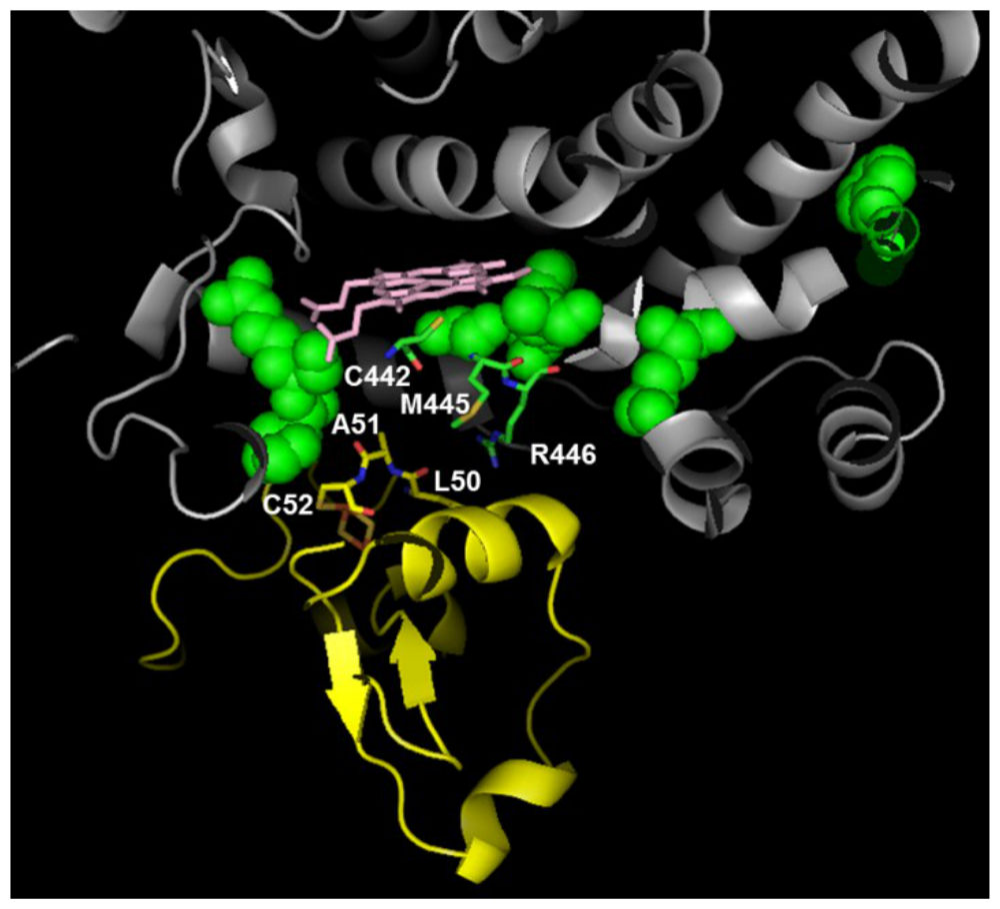
Molecular components of the catalytic network of the CYP3A4-adrenodoxin (Adx) complex. Adx is a small iron-sulfur protein that transfers electrons from NADPH to terminal CYP as part of the overall metabolic processing of substrates. We used the structural complex of this enzyme with eukaryotic CYP (CYP11A1) to model a CYP3A4-Adx complex. Selected molecular components of the modeled electron transfer pathway from the CYP3A4 heme iron to the Adx are shown, where the CYP3A4 unit is shown in gray, the Adx unit is shown in yellow, and the residues in the CYP3A4 catalytic networks are shown in green. The heme group is shown in pink, and the CYP3A4 C411 residue is shown in red. All residues involved in the predicted electron-transfer pathway are shown as sticks.

The NADPH-P450 reductase complex binds to CYP and enables the catalytic reaction by facilitating electron transfer from NADPH to the heme of P450 in a coupled two-step reaction. Although heavily studied, the details of the catalytic reaction mechanism are not clear [24-26]. In order to identify residues connected to the catalytic functionality we used the only currently available crystal structure of a complex between a eukaryotic CYP (CYP11A1) and its redox partner, adrenodoxin (Adx) [27]. We built reference models by superimposing the structure of the CYP11A1-Adx complex with CYP1A2, CYP2C9, CYP2D, and CYP3A4 structures to identify residues that could be associated with the catalytic reactions. Based on these structures, we identified residues that, by proximity, could be linked to catalysis in each CYP isoform. The cysteine residue, which connects to iron, is conserved among the isoforms, implying that the catalytic function is co-evolved and similar for different CYP isoforms. The positions E446/P447, E438/A439, and E461/V462 were predicted to belong to the catalytic network for CYP2D6, CYP2C9, and CYP1A2, respectively. For CYP3A4, the corresponding positions were M445/ R446. Figure 3 shows the predicted electron transfer pathway from the CYP3A4 heme iron to the Adx [2Fe-2S] cluster via side- and main-chain atoms, including C442, M445, and R446 from CYP3A4 and L50, A51, and C52 from Adx.

#### Dimerization networks

There is increasing experimental evidence indicating that CYP-CYP interactions occur among both the same and different CYP isoforms, resulting in the formation of both homo- and hetero-oligomers [18]. The oligomerization of CYPs significantly affects their capacity to function, substrate specificity, and potential for drug-drug interactions. To understand these functional consequences of oligomerization, it is essential to know how CYPs interact with each other. However, the architecture of these oligomer complexes and the functional consequences of oligomerization are still unclear. In the crystal structure of the CYP2C8 dimer, the F-G loop forms the dimer interface [28]. Although the crystallographic dimerization was facilitated by solubilizing the proteins, deleting the membrane anchor, and high protein concentrations, we used the F-G loop motif as a key signature for oligomerization. We identified co-evolutionary dimerization networks as those that contained residues that are physically on or close to the F-G loop. 

**Figure 4:**
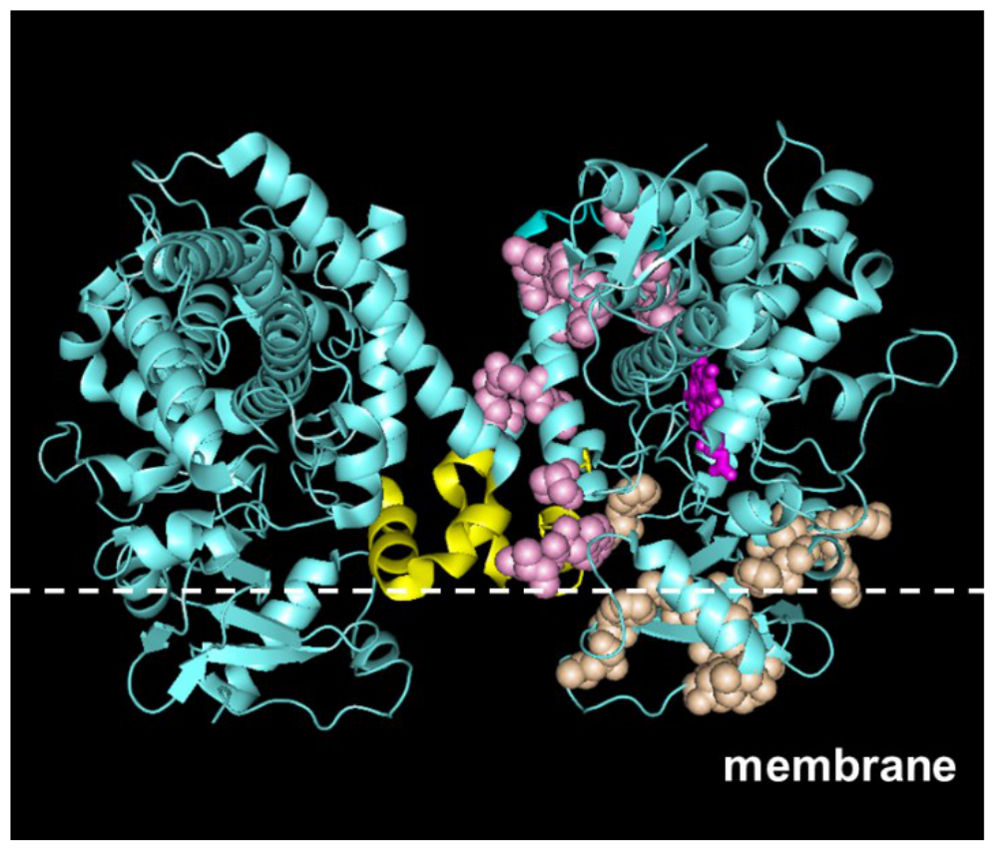
Functional networks of CYP2C9. Dimerization and membrane-binding networks were mapped on the crystal structure model of a CYP2C9 dimer (PDB code 1R9O). The dimerization network is shown in pink, the membrane-binding network is shown in orange, and the heme group is shown in magenta on the right monomer. The F-G loop is shown in yellow. The position of the membrane line was qualitatively positioned based on the location of the residues in the membrane-binding networks.

Although the F-G loop is a common motif, the dimerization network was absent in all isoforms except for the CYP2C subfamily. To visualize the impact of dimerization for other CYP2C isoforms, we constructed the CYP2C9-dimer based on the CYP2C8 crystal structure and mapped the dimerization network residues to the model. Figure 4 shows this dimer unit and its putative location and orientation in the membrane. The dimerization interface defined by our network was consistent with the existing crystal structure and cross-linking experiments, which highlighted F-G loop interactions in the CYP2C8 oligomer [18]. In our network of 14 residues, 10 residues were directly located at the dimerization interfaces and 4 residues were distal to the interface and indicative of a possible allosteric role for these residues. These putative allosteric sites are close to the substrate-binding sites, demonstrating a possible mechanistic explanation of how dimerization and allosteric regulation at the substrate-binding site alters CYP function.

#### Heme-binding networks

**Figure 5:**
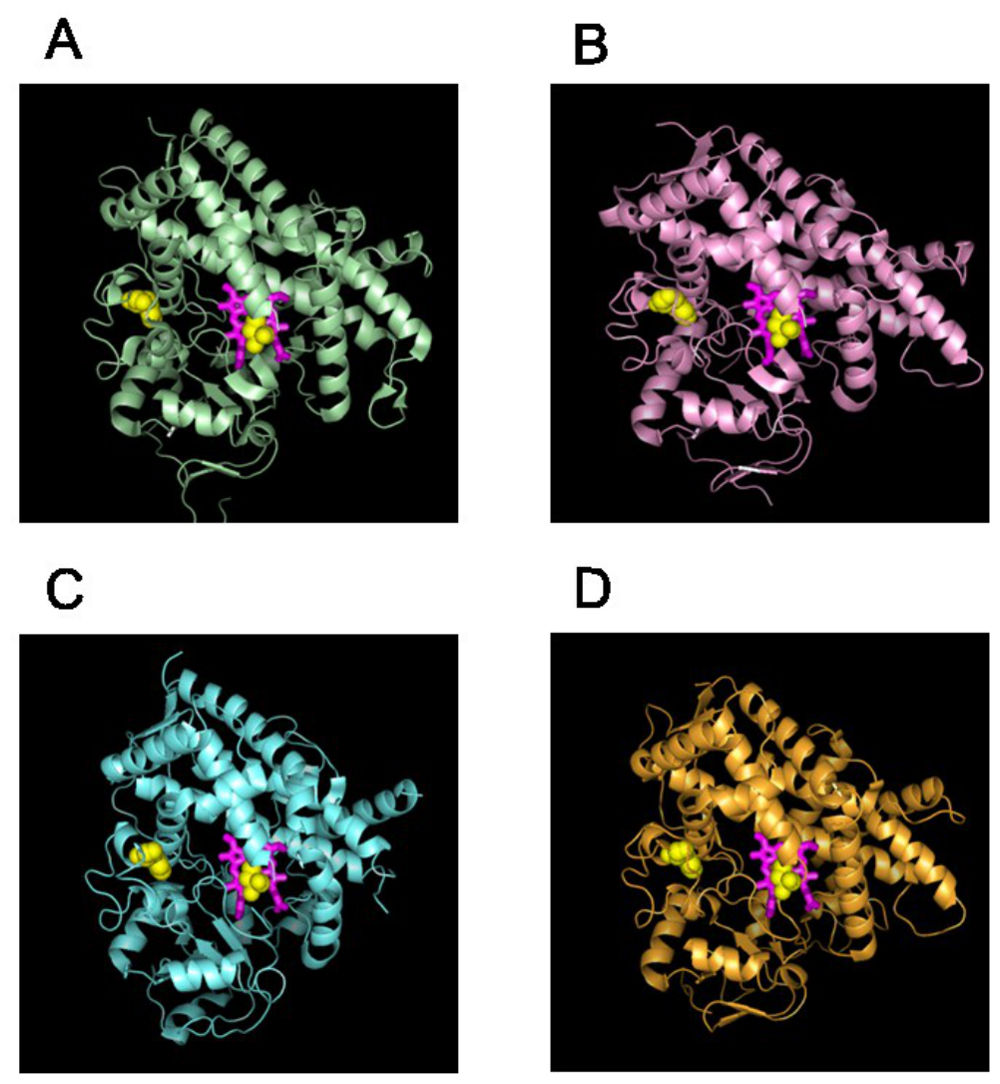
Co-evolved residues in the heme-binding networks. Residues identified as belonging to heme-binding networks were mapped to the molecular structures of CYP1A (PDB code 2HI4; *A*), CYP2C (PDB code 1R9O; *B*), CYP3A (PDB code 1W0E; *C*), and CYP2D (PDB code 2F9Q; *D*). The heme molecule is shown in stick representation, and residues belonging to the functional networks are shown as yellow spheres. Note that this network consists of both co-evolved residues that are in direct proximity to the heme as well as residues that are located distal to the heme.

When we mapped the last group of residues associated with co-evolution (Figure 1A) onto the protein structure, it was clear that they could be linked to the residues that bind the heme group. These residues form the heme-binding network and it is present in all four subfamilies. All networks contained both residues at and distal to the heme-binding site, with the latter constituting putative allosteric sites. Figure 5 shows the heme-binding network residues mapped to different CYP isoforms. For CYP3A4, the heme-binding network consisted of 21 residues, 11 residues of which were directly located at the heme-binding sites, leaving 10 residues as putative allosteric sites. 

### Allosteric effects of residues in the heme-binding networks

The co-evolved residue networks discussed above may have arisen from a multitude of causes. Here, we focused on identifying allosteric residues in these networks that could alter and regulate CYP metabolism. In particular, we wanted to investigate the molecular mechanisms behind Woods et al.’s recently reported results demonstrating that the metabolic products of the CYP3A4 substrate NR could be altered by ANF via an allosteric mechanism [7]. Our strategy was to link structural and dynamic information from the atomic descriptions of molecules and proteins with the sequence-based identification of co-evolved residue networks.

We used docking of NR to the CYP3A4 protein structure to search for key interacting residues at the substrate binding sites that may be important in determining the metabolic fate of the reactants. We used low-temperature ATD simulations to verify that these residues could exhibit long-range correlated motion with the putative allosteric residue in the network. We then used docking of the allosteric effector ANF to the protein to link binding of ANF to two of the putative allosteric residues identified in the heme-binding network by co-evolutionary analysis. These steps are detailed below, including an analysis of naturally occurring CYP3A4 mutants and proposed novel mutants that are hypothesized to allosterically regulate the metabolic transformation of NR. 

#### Interactions of NR reactants and products with CYP3A4

**Figure 6:**
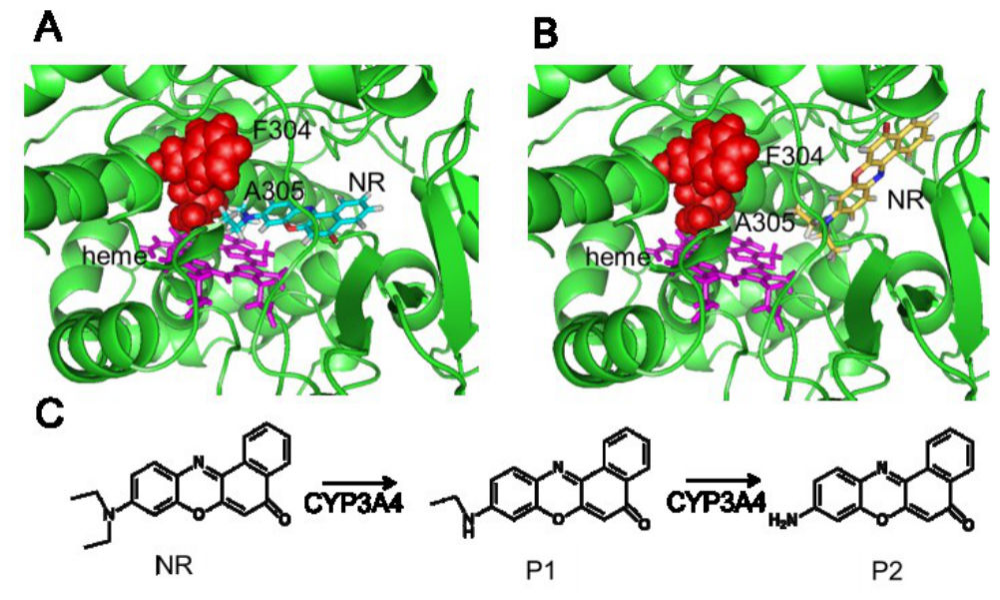
CYP3A4 interactions and metabolic fate of Nile red (NR). We docked the NR substrate to the CYP3A4 enzyme to identify how the substrate interacts with the heme group in the active site. The two top-scoring poses of NR are show in *A*, where the NR molecule is shown as sticks in orange (pose 1), and in *B*, where it is depicted in yellow (pose 2). Heme is shown as magenta sticks, residues F304 and A305 are shown as red spheres, and the rest of the enzyme is shown in green. *C*: possible reaction steps catalyzed by CYP3A4 in the conversion of NR to product *1* (P1) and product *2* (P2). The product ratio of P1 to P2 will depend on how the NR molecule and intermediate metabolites are positioned in the binding pocket.

We docked NR to CYP3A4 to determine key residues at or near substrate binding sites that would be susceptible to allosteric regulation. Figure 6, *A* and *B*, shows the two top-scoring docking poses for the modeled NR-CYP3A4 complex. Figure 6C shows the possible metabolic reactions of NR into product 1 (P1) and/or product 2 (P2). One pose (pose *1*; Figure 6A) has the NR sites of metabolism pointing toward the heme, whereas the other pose (pose *2*; Figure 6B) has these sites pointing away from the heme. When these poses were compared, pose *1* allows the NR-substrate to interact with the heme, which is not possible in pose *2*. We hypothesized that when NR adopts pose *1* in CYP3A4 the initial metabolic product is P1. If product P1 remains in this location, metabolism continues and P2 will be the major product. On the other hand, if P1 adopts pose 2, P1 will be the major product. The key residues that can “push” NR to leave pose *1* and adopt pose *2* are residues 301-306, which interact with pose *1* but not with pose *2*. Therefore, we hypothesized that mutations that change interactions between heme and residues 301-306 could allosterically alter CYP3A4 metabolism. To validate these docking poses, we performed 1 ns MD simulations for each initial docking pose by MD simulations. No significant structural fluctuations were observed in either case, as shown by the root mean square deviation in Figure S4. We further used the Molecular Mechanics/Poisson-Boltzmann Surface Area (MM/PBSA) method to estimate the binding free energies, which is -15.9 kcal/mol and -12.9 kcal/mol for pose1 and pose 2, respectively. The absolute binding free energies may not be accurate due to limited sample size, but they were consistent with the ranking for the top-scoring docking poses and validated these docking poses as plausible. 

#### Detection of long-range interactions in wild-type CYP3A4

**Figure 7:**
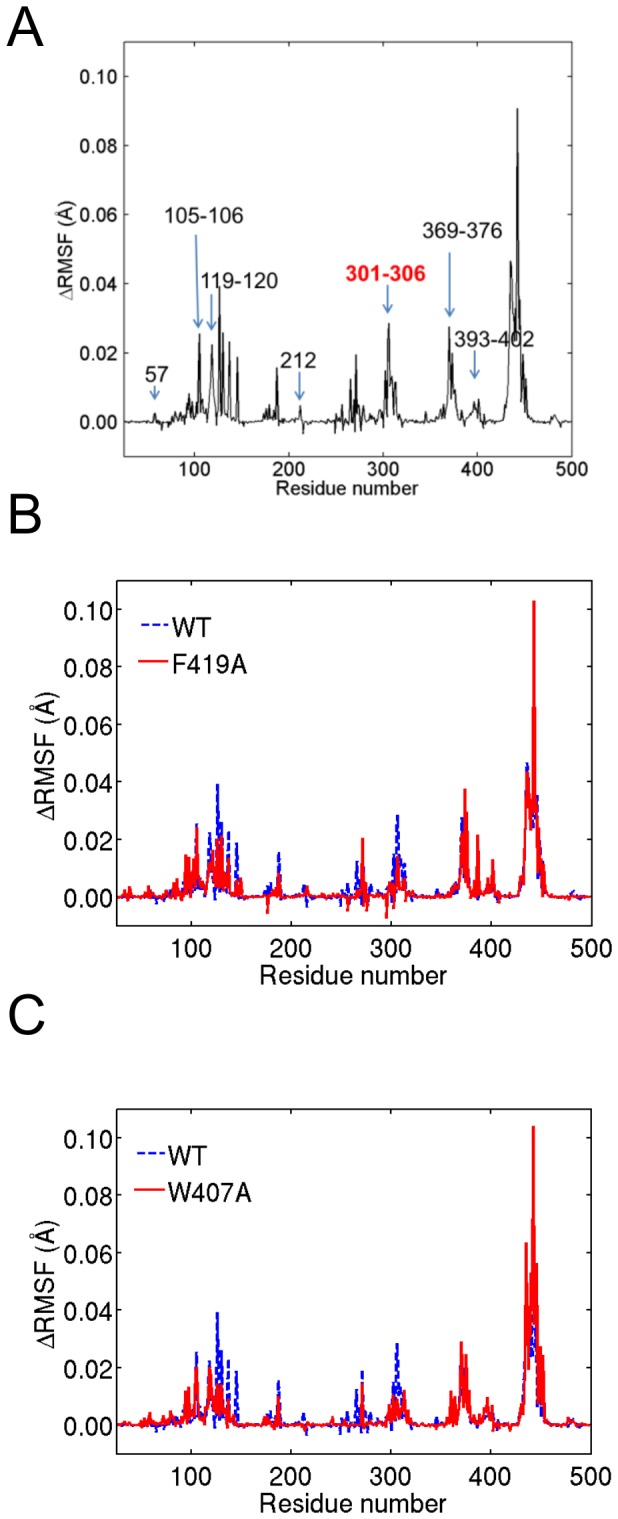
Residue-based root mean square fluctuations (ΔRMSFs). ΔRMSFs were calculated from low-temperature anisotropic thermal diffusion (ATD) simulations to identify residues that exhibit long-range correlated motions to a locally heated heme group in the CYP3A4 structure. *A*: WT CYP3A4 residues that interact with NR are labeled as numbers or number ranges and partially overlapped with the ΔRMSF peaks. Key residues 301-306, which provide the means to change the NR reaction products, are shown in red. *B* and *C*: ΔRMSFs for the F419A mutant compared with the WT (*B*) and for the W407A mutant compared with the WT (*C*). The sequence range that encompasses 301-306 is indicated by blue ovals. All residue numbers refer to the CYP3A4 sequence (PDB code 1W0E).

Next, we tested if simulations could detect long-range correlated motions and allosteric interactions between the heme and substrate-recognition residues. We performed ATD molecular dynamics simulations of wild-type (WT) CYP3A4 with the temperature of heme set to 300 K while the rest of the protein was kept at a low temperature of 10 K. Based on the correlated dissipation of heat through increased motion of residues that are sensitive to heme motions, residues that exhibit large fluctuations are coupled to heme fluctuations and, hence, have the capacity for long-range communication with heme. We then performed the simulation with the temperature of the whole protein set to 10K as the reference. We calculated root mean square fluctuation differences (ΔRMSF) for each residue between the two simulations to ascertain which residues exhibit large fluctuations during the simulation. Based on the NR-CYP3A4 docking results, the following 19 CYP3A4 residues interacted with the NR substrate: 57, 105-106, 119-120, 212, 215-216, 301, 304-305, 369-374, 393, and 395. Figure 7A shows that 17 of the 19 substrate-interacting residues coincided with peaks in the ΔRMSF distribution, indicating that the ATD-generated correlated motion identified possible long-range communications between heme and substrate-binding residues.

#### Detection of long-range interactions in naturally occurring CYP3A4 mutants

We further explored the hypothesis that residues 301-306 are key in affecting allosteric regulation. In particular, we investigated whether the changes of interactions between heme and residues 301-306 by a known mutation were correlated with changes in CYP3A4 metabolism. We tested four natural occurring mutants M445T [29], F189S [29], I118V [30], and L293P [29] by performing ATD simulations for the mutants and comparing the ΔRMSFs with those of the WT to see if the mutation significantly changed the residue fluctuation of residues 301-306. We analyzed two trajectories for each mutation. The simulation results for all four mutants are shown in Figure S5-S6.

M445T mutations naturally occur in Caucasians with an allelic frequency of 4%. Previous work has showed that the M445T enzyme metabolizes drugs such as testosterone, chlorpyrifos, and progesterone similar to the WT CYP3A4 enzyme [29]. Our ATD simulation with the M445T mutant construct showed no significant ΔRMSF differences for residues 301-306 compared with the WT enzyme. This is consistent with the notion that the M445T mutant does not alter the metabolic fate of its substrates through an allosteric mechanism affecting heme binding. 

F189S and L293P mutations naturally occur with an allelic frequency of 2% in Caucasians and Asians, respectively. Compared with the WT, both mutants significantly change the turnover numbers for testosterone and chlorpyrifos [29]. Our ATD simulation detected significantly changed fluctuation of residues 301-306 for both F189S and L293P compared with the WT, indicative of the experimentally observed altered metabolism for these mutant enzymes. Interestingly, the experimental data showed that F189S had lower turnover numbers for testosterone and chlorpyrifos, whereas L293P exhibited higher turnover numbers for both substrates. One of the reasons that our results cannot predict the turnover numbers is because our simulations can only detect changes in fluctuation at the active sites, but not how the mutation potentially alters the active site conformation, which will be critical for predicting turnover numbers.

I118V is a naturally occurring CYP3A4 mutant occurring in the Han Chinese population with an allelic frequency of 3.3%. This mutation is associated with altered CYP3A4 metabolism and multiple new metabolic products for anandamide were observed for the I118V mutant enzyme [30]. Our ATD simulations showed significantly changed fluctuations of residues 301-306 for the I118V mutant, indicative of a possible altered metabolic fate for compounds metabolized by this CYP3A4 mutant. Interestingly, our two simulations showed increased and decreased fluctuation respectively. Furthermore, I118V has been reported to lead to the formation of several new metabolites [30], indicative of multiple conformational states of I118V leading to the formation of different metabolites. This confirms that our method predicts possible altered activity by allosteric mutations, but should not be used to predict the nature of the altered activity. 

In summary, for all of the above naturally occurring mutants we measured ΔRMSF changes for residues 301-306 when altered metabolic behavior was observed experimentally. This implies that these residues will be key residues in detecting allosteric mutations that affect CYP3A4 metabolism. 

#### Interaction of the allosteric modulator ANF with CYP3A4

To identify allosteric mutations that may affect the metabolic behavior of CYP3A4, we first predicted possible binding sites of the known allosteric modulator ANF. We docked ANF to CYP3A4, and the top-scoring docking poses are shown in Figure S7. Among the top 10 docking poses, 6 poses were located at the substrate-binding site, which is consistent with ANF also being a substrate of CYP3A4 [7]. On the other hand, poses *4*, *6*, *8*, and *10* were not located at or near the substrate-binding pocket. We performed ATD simulations for the bound ANF-CYP3A4 complex for each of the four docked poses and compared the results with unbound CYP3A4 simulations. The fluctuations of residues 301-306 decreased for poses *4*, *6*, and *8*, whereas there were no significant changes for pose *10*. Therefore, we predicted that poses *4*, *6*, and *8* are potential ANF allosteric binding sites, whereas pose *10* is not. A recent allosteric effector binding-site study, using fluorescence resonance energy transfer and fluorol-7GA as a model substrate [31], also implicated peripheral sites similar to our poses *4* and *8*. 

#### Allosteric mutant design

Our final step was to propose allosteric mutants based on the binding sites defined by docking poses *4*, *6*, and *8*. Interestingly, the naturally occurring mutation L293P falls into the binding site of pose *6*. As we discussed in the previous section, L293P significantly changed the fluctuation of key residues 301-306 computationally and is known experimentally to allosterically affect CYP3A4 metabolism [29]. To demonstrate that the mutations would not randomly perturb the dynamics of the key residues due to nonspecific disruption of the protein structure, we performed control simulations for two additional mutants, F219A and R418A, predicted to have no allosteric effect. Both simulations showed no significant changes of the ΔRMSF of the key residues 301-306, as shown in Figure S8.

To design mutations at the binding sites of poses *4* and *8*, we examined candidates belonging to the heme-binding network and identified F419 and W407 as potential allosteric effector binding sites. We created enzyme structures of both F419 and W407 converted to alanine and performed ATD simulations using the structures to ascertain their impact on fluctuations of key residues 301-306. Figure 7, B and C, shows that the ΔRMSF for the alanine mutations significantly reduced fluctuations compared with the WT of key residues 301-306 for both F419A and W407A. We further performed the second trajectory for both mutants and observed similar fluctuation changes, as shown in Figure S9. These results suggest that mutation of these residues will alter the dynamic behaviors of the key residues and, thus, change CYP3A4 metabolism, similar to what was observed for the naturally occurring L293P mutant. 

## Discussion

We identified four CYP mechanical and functional networks based on a co-evolutionary analysis. We further hypothesized that residues in these networks that are spatially distant to actives sites, or other regions important for protein function, allosterically regulate activity of the enzymes. Finally, we computationally tested this hypothesis using low-temperature ATD simulations and showed that mutation of F419 and W407 in the heme-binding network would allosterically affect key CYP3A4 residues involved in determining metabolic fate.

Among the four functional networks, none included residues in the substrate-binding pocket, suggesting that substrate-binding residues are not co-evolved but instead are specific for each species. In other words, the substrate recognition sites also determine substrate specificity. Recently, experiments have shown that CYP metabolism in animal models such as mouse, rat, or rabbit are significantly different from that of human [32]. In line with this observation, our results suggest that the substrate recognition sites have been selectively mutated during evolution, a natural consequence of species-specific environments and chemical/toxin exposure during evolution. 

A single N-terminal transmembrane α helix anchors the CYP enzyme in the membrane. The interaction of an enzyme with the membrane directly affects how the substrate gains access to the active site. The exact membrane binding residues for each CYP isoform remain unknown despite extensive experimental work, e.g., probing the orientation of the enzyme in the membrane using the heme tilt angle with respect to the membrane [33], the height of the globular domain above the lipid bilayer [34], etc. However, many of these experiments are not consistent with each other, implying that some isoforms may adopt different orientations in the membrane and that different CYPs may have different orientational preferences. Our co-evolutionary analysis suggested that the CYP3A, CYP1A, CYP2D, and CYP2C subfamilies adopt different orientations in the membrane. The CYP3A membrane network included a wide range of residues, indicative of both specific and variable isoform orientations in the membrane. Consistent with our analysis, the CYP3A and CYP2D isoforms, such as CYP3A4 and CYP2D6, are also known to be substrate promiscuous and included both lipo-soluble and water-soluble substrates. This substrate promiscuity is a direct consequence of the ability of the enzyme to adapt different orientations in the membrane and allow access to its active site from both the cytosol and membrane-bound fraction. On the other hand, the main substrates for the isoforms in the CYP1A family, such as CYP1A2, are planar amines and amides, which are soluble in water, whereas the substrates for CYP2C9 are lipophilic molecules. The membrane-binding networks we detected for the CYP1A and CYP2C families showed two distinct and different orientations. Whereas the access channel for CYP1A allowed substrates to be processed from the cytosol, the CYP2C access channel was clearly associated with the membrane-bound fraction. The identification of a co-evolved membrane network provides a novel angle to study CYP-membrane interactions. As the methodology is generally applicable to any protein, a co-evolutionary analysis could be used to functionally characterize other membrane-bound proteins.

We identified a dimerization network associated with the CYP2C family. Our location of this network is commensurate with the recent work by Hu et al. [18], which identified the F-G loop region as the CYP2C8 dimerization interface. Interestingly, we only detected the dimerization network for the CYP2C subfamily and not for the CYP3A and CYP1A subfamilies. This raised the question whether dimerization is a unique feature for the CYP2C subfamily or whether it is a broader property among other select CYP isoforms. As some members in the CYP1A and CYP3A subfamilies have shown a tendency to form dimers [35] and the dimerization network detected here was based on an analysis of co-evolved residues, we cannot exclude that other CYP isoforms could oligomerize based on physiochemical features not captured by residue co-evolution.

In all the networks we characterized, it was evident that some residues were closely associated with a particular facet of CYP metabolism by their close physical proximity to regions of the protein associated with these functions. Importantly, these residues were also connected, via the co-evolution analysis, with residues that were physically distal to these functions and hypothesized to be involved in allosteric regulation. These distal residues would then be associated with allosteric sites that could be perturbed by other molecules. For example, in CYP3A4, 4 of 14 residues in the dimerization network were not located at the dimer interface, and 11 of 21 residues in the heme-binding network could be associated with possible allosteric sites. We hypothesized that these residues have the ability to regulate CYP function via multiple allosteric mechanisms. We used the heme-binding network as an example to test this hypothesis based on Woods et al.’s report that the addition of ANF allosterically alters the reaction products of NR when metabolized by CYP3A4 [7]. We performed ATD simulations for the WT strain and for naturally occurring mutants to confirm that these mutations could allosterically affect the behavior of the key residues involved in NR binding and metabolism. We further docked the allosteric effector ANF to CYP3A4 and showed that residues F419 and W407, which are not directly involved in heme binding, are located in the most favorable docking pockets for ANF. Finally, ATD simulations using F419A and W407A mutants showed that these mutations could allosterically affect the key NR-metabolizing residues. In this way, we provided a structural and dynamic link between residues F419 and W407 predicted by co-evolutionary analysis and their allosteric regulation of CYP3A4 metabolism. Although additional experimental work will be needed to further evaluate the role of allosteric regulation among the networks described here, the computational framework provides a capability to understand and generate testable hypotheses for the allosteric regulation of protein function. 

## Methods

### Co-evolutionary analysis

Multiple sequence alignment was performed with MUSCLE [36] and modified with Gblocks [37] for each CYP family. We downloaded and compiled Maximal SubTree (MST) software [14] for allosteric network prediction. The number of *maximal subtrees* is the largest subtree with the co-evolved residue at a given sequence position and is calculated for each position in the multiple sequence alignment. MST assigned this number of each position to be the rank of this position. The smaller the rank, the stronger the evolutionary pressure is at this position. To identify networks of co-evolved residues, MST first identified seed positions, which correspond to sites where conservation of subtrees is sufficiently stable with respect to a mean rank. To evaluate the co-evolution of each pair of seed positions, a correspondence matrix was constructed based on the *maximal subtrees* associated with each position, and a co-evolution score was calculated for each pair of seed positions. Positions with similar co-evolution scores were clustered together using the algorithm implemented in the MST tool and allowed us to construct networks of co-evolved residues based on cluster membership. 

### Molecular modeling

We docked small molecules to proteins using PatchDock [38]. The structures of CYP3A4, CYP1A2, CYP2C9, and CYP2D6 were abstracted from the crystal structures (PDB code 1W0E [39], 2HI4 [40], 1R9O [41], and 2F9Q [42], respectively). We added and modeled missing residues in these structures using SWISS-MODEL [43]. The access channel analysis was performed using MOLEonline 2.0 [44]. 

### ATD simulations

We performed ATD simulations [13] using the CHARMM27 force field [45]. The force field for heme, as well as the corresponding iron and sulphur related parameters, were obtained from Bathelt et al. [46]. These parameters are based on QM/MM calculations and have been used in several CYP MD simulations studies [47,48]. The force field parameters for NR and ANF were generated using SwissParam [49]. The minimized complex was cooled to 10 K during 1 ns, until the overall RMSF in atom positions was <0.05 Å. In this structure, the side chain of a selected residue was coupled to a thermal heat bath at 300 K, whereas the rest of the system was left uncoupled. The receptor complexes were positionally restrained by a harmonic potential acting on the backbone CA atoms (5 kcal mol^-1^ Å^-2^) and on atoms with a solvent accessible surface area of >10 Å^2^.

To implement the ATD simulations, we first validated our protocol by testing it on PSD-95 and verifying that our results reproduced the published results [13]. With the protocol confirmed, we performed ATD simulation for CYP3A4. We locally heated the heme group and observed the heat propagation to residues far away from the heme. The residues through which heat and correlated structural fluctuations occur were designated as the heme-binding network and comprised both residues close to heme and the active site as well as residues distant to these sites and located on the protein surface. Unlike values reported by Ota et al. [13], our RMSFs displayed relatively lower absolute values. This was partly due to our calculations including a correction for the background noise, as well as our choice of heating of the relatively more strongly bound heme-group rather than residue-heating employed by Ota et al. These factors both contributed to the observed slow heat propagation and low RMSFs.

Restraints on surface atoms were introduced by Ota et al. [13] to avoid energy transmission toward the solvent and were similarly implemented here. Given the restraints on surface atoms and the short simulation time, we gauged the solvent influence to be minimal, and, hence, we excluded all water molecules. To prevent the typical large conformational changes that are associated with gas phase equilibration, we added restraints on backbone atoms and solvent-accessible atoms. ATD molecular dynamics simulations using velocity Verlet dynamics were performed for 10 ps. The diffusion of heat was detected by root-mean-square deviation time series of the amino acid side chains. 

## Supporting Information

Figure S1 **Networks detected by co-evolutionary analysis for three different cytochrome P450 (CYP) isoforms: CYP3A (*A*), CYP1A (*B*), and CYP2D (*C*).** The heat map shows the co-evolutionary score for each pair of residues that was tagged as evolving together above the evolutionary noise. Not all residues in the sequences are shown and the order of the residues was determined via clustering based on the co-evolutionary score. The residue clustering identified three primary groupings, which we identified as a membrane-binding network, a catalytic network, and a heme-binding network. Although the networks are separable by their primary function, crosstalk can occur between these networks and functions as evident from the off-diagonal elements. Each pairwise entry was color coded from high (red) to low (blue) co-evolutionary scores.
(DOC)

Figure S2 **Validation for prediction of membrane binding network for CYP2C.** (A) Comparison of predicted and experimental models of embedding of CYP2C protein into the membrane. The predicted membrane upper layer is shown in planes. Our predicted model is shown in green, experimental models for CYP2B4 and CYP2C5 are shown in red and yellow, respectively. (B) RMSDs of the CYP2C9 simulation using implicit solvent model.
(DOC)

Figure S3 **Access channel for different CYP isoforms: CYP1A (*A*), CYP2C (*B*), CYP3A (*C*), and CYP2D (*D*).** In *A-D*, the channel is shown as a solid-colored volume that provides a path for substrates to move from the exterior to the interior of the proteins. In B-C, two access channels are shown in different colors. The membrane-binding network is indicated by spheres and provides a spatial reference to which channels are open to the solvent and/or the membrane-bound fraction.
(DOC)

Figure S4 **RMSDs for simulations on two NR docking poses on CYP3A4.** (A) Pose 1; (B) Pose 2. The RMSDs for protein and ligand are shown in black and red lines, respectively.
(DOC)

Figure S5 **Comparison of root mean square fluctuations (ΔRMSFs) from anisotropic thermal diffusion simulation of naturally occurring mutants with wild-type (WT) CYP3A4: M445T (*A*), F189S (*B*), I118V (*C*), and L293P (*D*).** Key residues 301-306 are highlighted by blue ovals. All residue numbers refer to the CYP3A4 sequence (PDB code 1W0E).
(DOC)

Figure S6 **The second trajectories of comparison of root mean square fluctuations (ΔRMSFs) from anisotropic thermal diffusion simulation of naturally occurring mutants with wild-type (WT) CYP3A4: M445T (**A**), F189S (**B**), I118V (**C**), and L293P (**D**).** Key residues 301-306 are highlighted by blue ovals. All residue numbers refer to the CYP3A4 sequence (PDB code 1W0E).
(DOC)

Figure S7 **Docking poses of the allosteric effector α-naphthoflavone (ANF) to CYP3A4 (PDB code 1W0E).** The top-ranking poses at the allosteric sites are numbered *4*, *6*, *8*, and *10* and are shown as thicker sticks. ANF molecules docked and positioned at the heme group are shown as thin sticks. The heme group is shown as a red stick model.
(DOC)

Figure S8 **Comparison of root mean square fluctuations (ΔRMSFs) from anisotropic thermal diffusion simulation of control mutants with wild-type (WT) CYP3A4: F219A (**A**) and R418A (**B**).** Key residues 301-306 are highlighted by blue ovals. All residue numbers refer to the CYP3A4 sequence (PDB code 1W0E).
(DOC)

Figure S9 **The ΔRMSFs of second trajectories for two mutants.** (A) F419A mutant compared with the WT; (B) W407A mutant compared with the WT. The sequence range that encompasses 301-306 is indicated by blue ovals. All residue numbers refer to the CYP3A4 sequence (PDB code 1W0E).
(DOC)
