# Supplementary material for: Identifying Cytochrome P450 Functional Networks and Their Allosteric Regulatory Elements
Source: PLoS One. 2013 Dec 3;8(12):e81980. doi: 10.1371/journal.pone.0081980 (PMC3849357; doi:10.1371/journal.pone.0081980)
Supplement: Figure S1 — Networks detected by co-evolutionary analysis for three different cytochrome P450 (CYP) isoforms: CYP3A (A), CYP1A (B), and CYP2D (C). The heat map shows the co-evolutionary score for each pair of residues that was tagged as evolving together above the evolutionary noise. Not all residues in the sequences are shown and the order of the residues was determined via clustering based on the co-evolutionary score. The residue clustering identified three primary groupings, which we identified as a membrane-binding network, a catalytic network, and a heme-binding network. Although the networks are separable by their primary function, crosstalk can occur between these networks and functions as evident from the off-diagonal elements. Each pairwise entry was color coded from high (red) to low (blue) co-evolutionary scores. (DOC) [file pone.0081980.s001.doc]

**
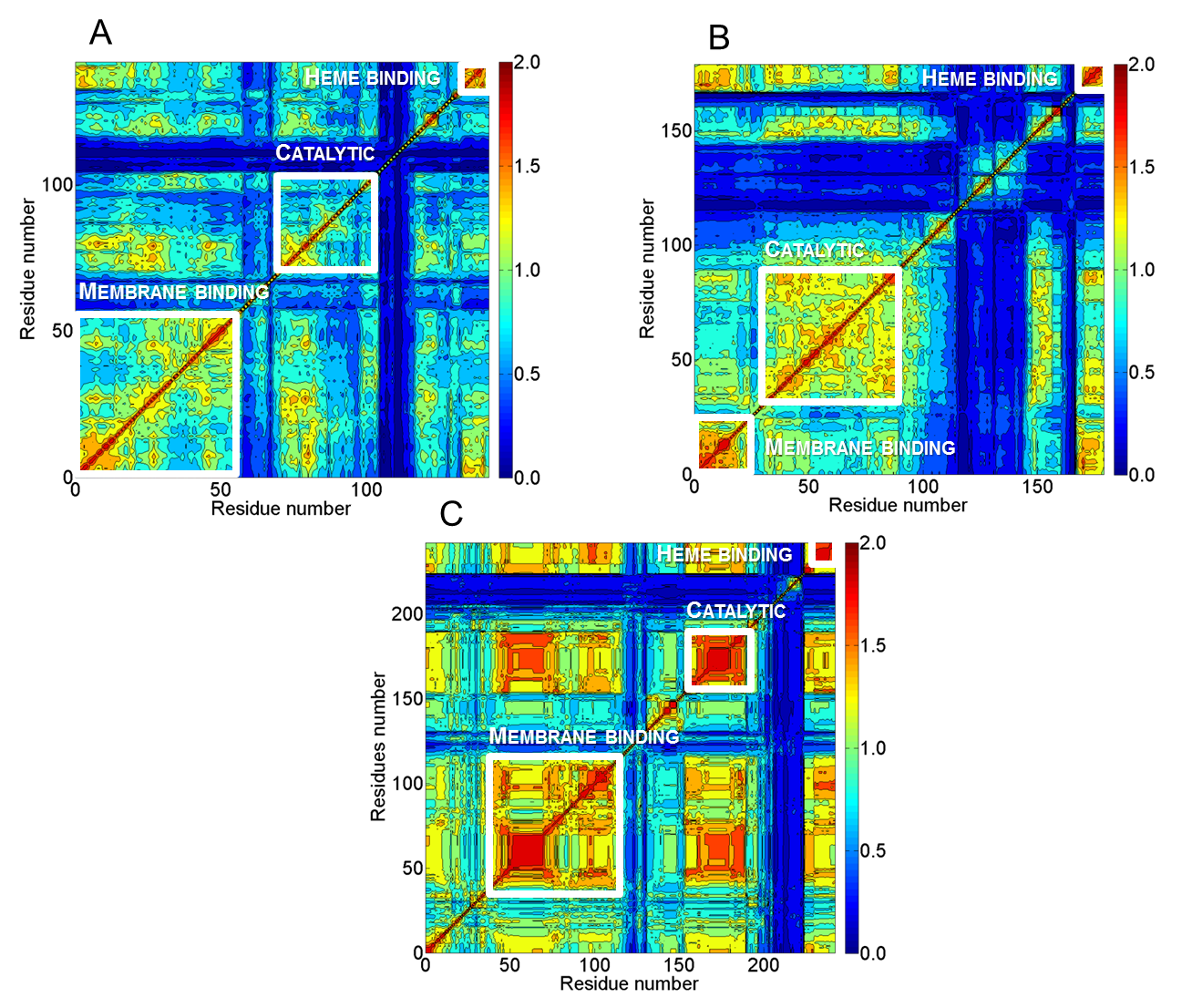
**

**Figure S1.** Networks detected by co-evolutionary analysis for three different cytochrome P450 (CYP) isoforms: CYP3A (*A*), CYP1A (*B*), and CYP2D (*C*). The heat map shows the co-evolutionary score for each pair of residues that was tagged as evolving together above the evolutionary noise. Not all residues in the sequences are shown and the order of the residues was determined via clustering based on the co-evolutionary score. The residue clustering identified three primary groupings, which we identified as a membrane-binding network, a catalytic network, and a heme-binding network. Although the networks are separable by their primary function, crosstalk can occur between these networks and functions as evident from the off-diagonal elements. Each pairwise entry was color coded from high (red) to low (blue) co-evolutionary scores.
