# Supplementary material for: Identifying Cytochrome P450 Functional Networks and Their Allosteric Regulatory Elements
Source: PLoS One. 2013 Dec 3;8(12):e81980. doi: 10.1371/journal.pone.0081980 (PMC3849357; doi:10.1371/journal.pone.0081980)
Supplement: Figure S2 — Validation for prediction of membrane binding network for CYP2C. (A) Comparison of predicted and experimental models of embedding of CYP2C protein into the membrane. The predicted membrane upper layer is shown in planes. Our predicted model is shown in green, experimental models for CYP2B4 and CYP2C5 are shown in red and yellow, respectively. (B) RMSDs of the CYP2C9 simulation using implicit solvent model. (DOC) [file pone.0081980.s002.doc]

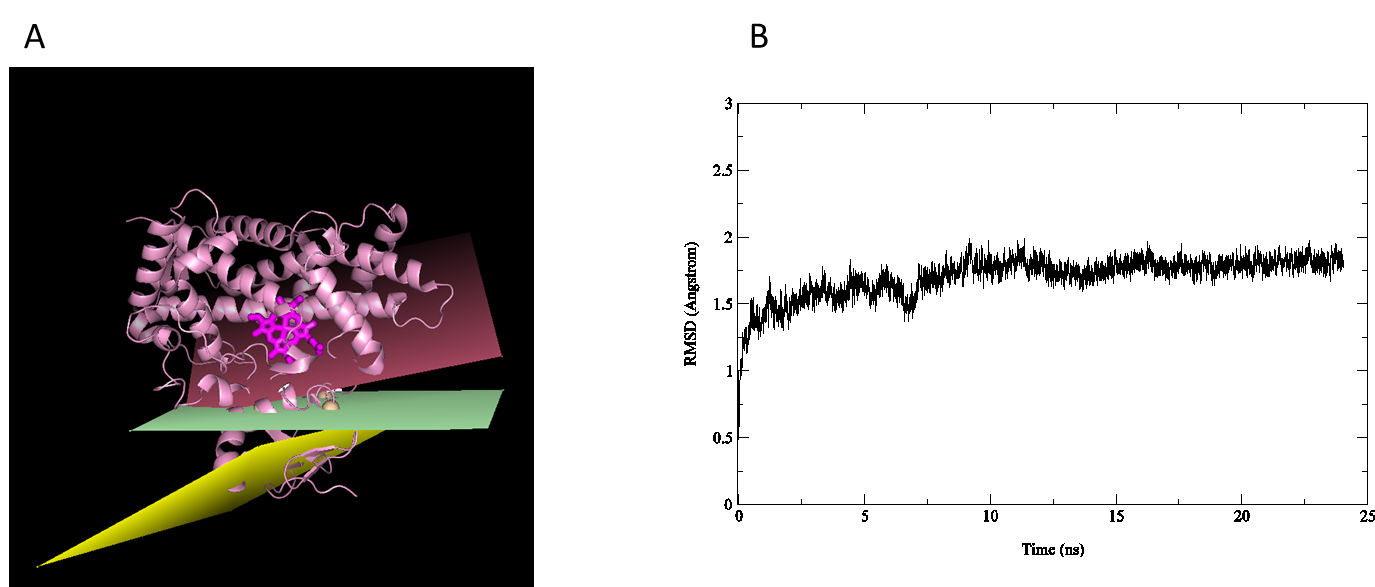


**Figure S2.** Validation for prediction of membrane binding network for CYP2C. (A) Comparison of predicted and experimental models of embedding of CYP2C protein into the membrane. The predicted membrane upper layer is shown in planes. Our predicted model is shown in green, experimental models for CYP2B4 and CYP2C5 are shown in red and yellow, respectively. (B) RMSDs of the CYP2C9 simulation using implicit solvent model.
