# Supplementary material for: Identifying Cytochrome P450 Functional Networks and Their Allosteric Regulatory Elements
Source: PLoS One. 2013 Dec 3;8(12):e81980. doi: 10.1371/journal.pone.0081980 (PMC3849357; doi:10.1371/journal.pone.0081980)
Supplement: Figure S3 — Access channel for different CYP isoforms: CYP1A (A), CYP2C (B), CYP3A (C), and CYP2D (D). In A-D, the channel is shown as a solid-colored volume that provides a path for substrates to move from the exterior to the interior of the proteins. In B-C, two access channels are shown in different colors. The membrane-binding network is indicated by spheres and provides a spatial reference to which channels are open to the solvent and/or the membrane-bound fraction. (DOC) [file pone.0081980.s003.doc]

**
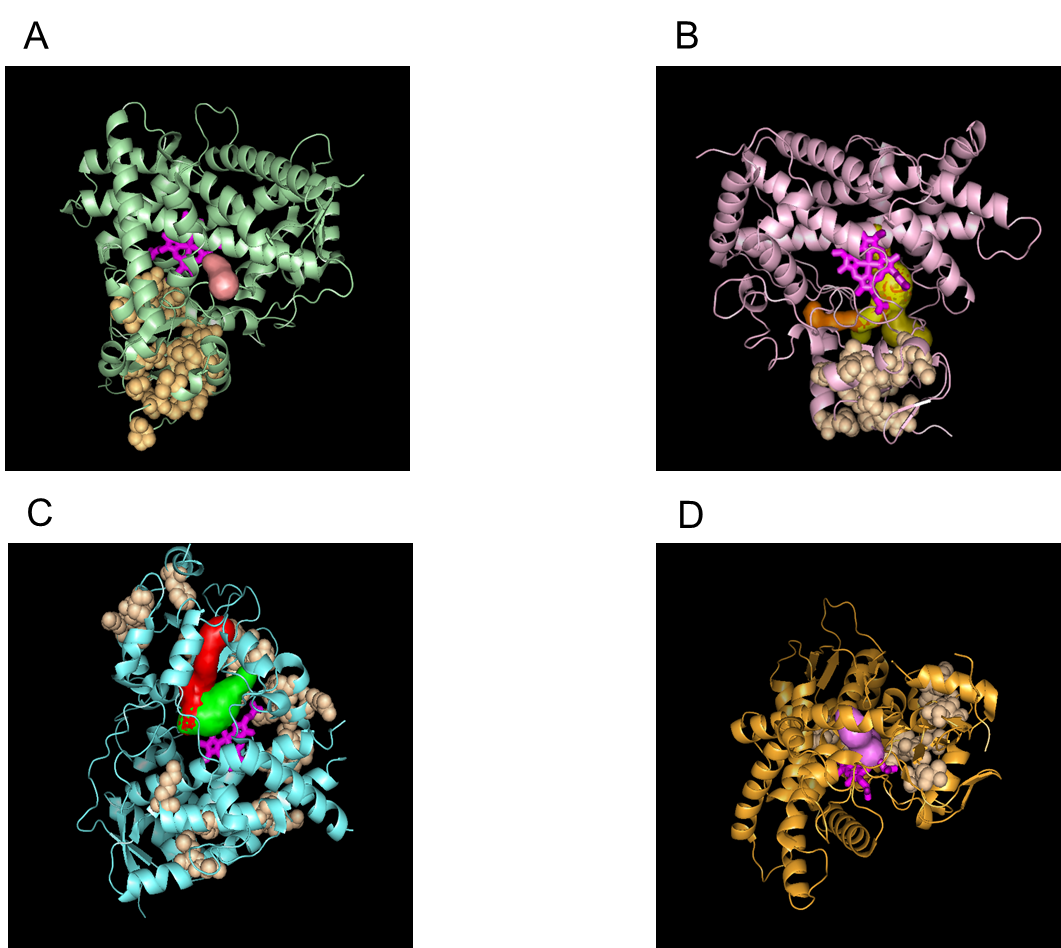
**

**Figure S3.** Access channel for different CYP isoforms: CYP1A (*A*), CYP2C (*B*), CYP3A (*C*), and CYP2D (*D*). In *A-D*, the channel is shown as a solid-colored volume that provides a path for substrates to move from the exterior to the interior of the proteins. In B-C, two access channels are shown in different colors. The membrane-binding network is indicated by spheres and provides a spatial reference to which channels are open to the solvent and/or the membrane-bound fraction.
