# Supplementary material for: Identifying Cytochrome P450 Functional Networks and Their Allosteric Regulatory Elements
Source: PLoS One. 2013 Dec 3;8(12):e81980. doi: 10.1371/journal.pone.0081980 (PMC3849357; doi:10.1371/journal.pone.0081980)
Supplement: Figure S4 — RMSDs for simulations on two NR docking poses on CYP3A4. (A) Pose 1; (B) Pose 2. The RMSDs for protein and ligand are shown in black and red lines, respectively. (DOC) [file pone.0081980.s004.doc]

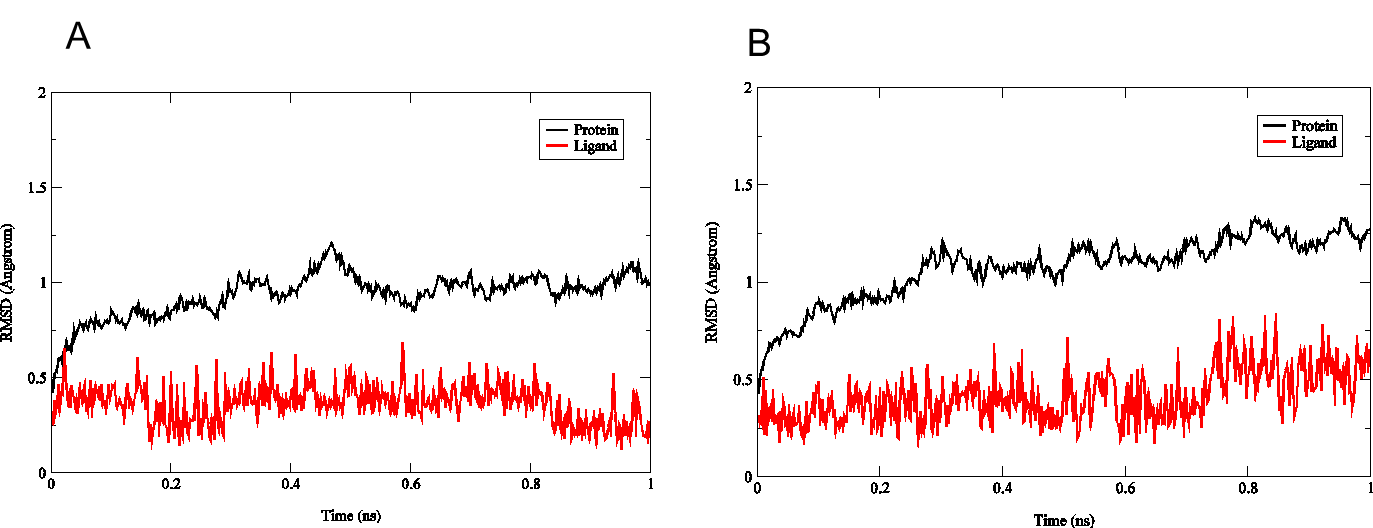


**Figure S4.** RMSDs for simulations on two NR docking poses on CYP3A4. (A) Pose 1; (B) Pose 2. The RMSDs for protein and ligand are shown in black and red lines, respectively.
