# Supplementary material for: Identifying Cytochrome P450 Functional Networks and Their Allosteric Regulatory Elements
Source: PLoS One. 2013 Dec 3;8(12):e81980. doi: 10.1371/journal.pone.0081980 (PMC3849357; doi:10.1371/journal.pone.0081980)
Supplement: Figure S6 — The second trajectories of comparison of root mean square fluctuations (ΔRMSFs) from anisotropic thermal diffusion simulation of naturally occurring mutants with wild-type (WT) CYP3A4: M445T (A), F189S (B), I118V (C), and L293P (D). Key residues 301-306 are highlighted by blue ovals. All residue numbers refer to the CYP3A4 sequence (PDB code 1W0E). (DOC) [file pone.0081980.s006.doc]

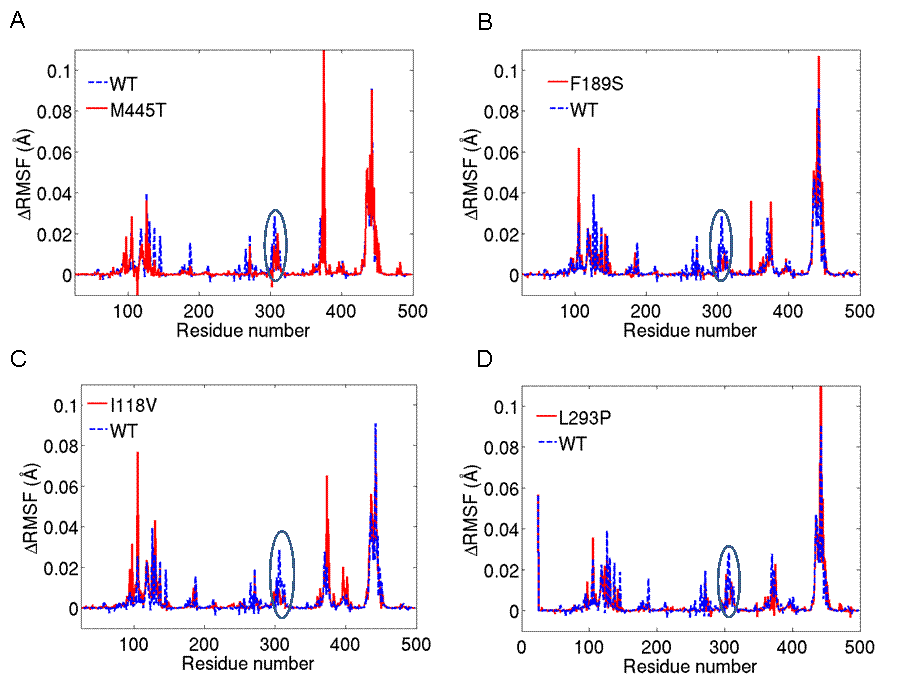


**Figure S6.** The second trajectories of comparison of root mean square fluctuations (ΔRMSFs) from anisotropic thermal diffusion simulation of naturally occurring mutants with wild-type (WT) CYP3A4: M445T (*A*), F189S (*B*), I118V (*C*), and L293P (*D*). Key residues 301-306 are highlighted by blue ovals. All residue numbers refer to the CYP3A4 sequence (PDB code 1W0E).
