# Supplementary material for: Identifying Cytochrome P450 Functional Networks and Their Allosteric Regulatory Elements
Source: PLoS One. 2013 Dec 3;8(12):e81980. doi: 10.1371/journal.pone.0081980 (PMC3849357; doi:10.1371/journal.pone.0081980)
Supplement: Figure S7 — Docking poses of the allosteric effector α-naphthoflavone (ANF) to CYP3A4 (PDB code 1W0E). The top-ranking poses at the allosteric sites are numbered 4, 6, 8, and 10 and are shown as thicker sticks. ANF molecules docked and positioned at the heme group are shown as thin sticks. The heme group is shown as a red stick model. (DOC) [file pone.0081980.s007.doc]

**
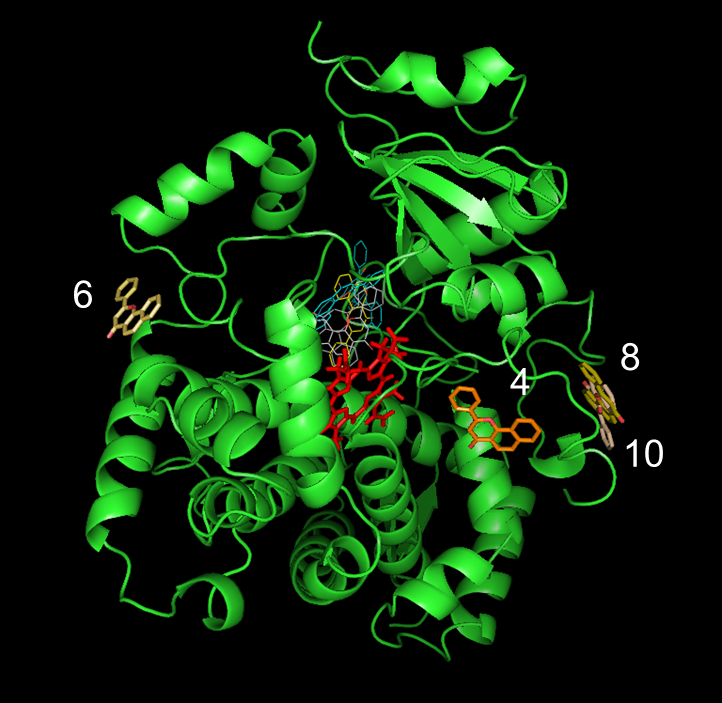
**

**Figure S7.** Docking poses of the allosteric effector α-naphthoflavone (ANF) to CYP3A4 (PDB code 1W0E). The top-ranking poses at the allosteric sites are numbered *4*, *6*, *8*, and *10* and are shown as thicker sticks. ANF molecules docked and positioned at the heme group are shown as thin sticks. The heme group is shown as a red stick model.
