# Supplementary material for: Identifying Cytochrome P450 Functional Networks and Their Allosteric Regulatory Elements
Source: PLoS One. 2013 Dec 3;8(12):e81980. doi: 10.1371/journal.pone.0081980 (PMC3849357; doi:10.1371/journal.pone.0081980)
Supplement: Figure S8 — Comparison of root mean square fluctuations (ΔRMSFs) from anisotropic thermal diffusion simulation of control mutants with wild-type (WT) CYP3A4: F219A (A) and R418A (B). Key residues 301-306 are highlighted by blue ovals. All residue numbers refer to the CYP3A4 sequence (PDB code 1W0E). (DOC) [file pone.0081980.s008.doc]

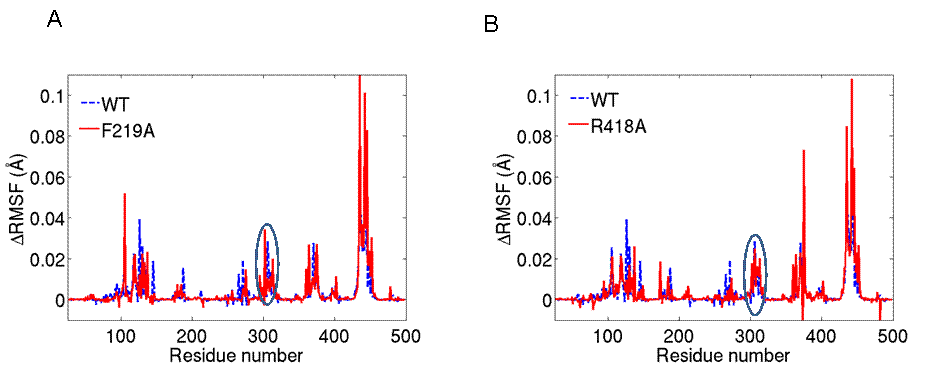


**Figure S8.** Comparison of root mean square fluctuations (ΔRMSFs) from anisotropic thermal diffusion simulation of control mutants with wild-type (WT) CYP3A4: F219A (*A*) and R418A (*B*). Key residues 301-306 are highlighted by blue ovals. All residue numbers refer to the CYP3A4 sequence (PDB code 1W0E).
