# Supplementary material for: Identifying Cytochrome P450 Functional Networks and Their Allosteric Regulatory Elements
Source: PLoS One. 2013 Dec 3;8(12):e81980. doi: 10.1371/journal.pone.0081980 (PMC3849357; doi:10.1371/journal.pone.0081980)
Supplement: Figure S9 — The ΔRMSFs of second trajectories for two mutants. (A) F419A mutant compared with the WT; (B) W407A mutant compared with the WT. The sequence range that encompasses 301-306 is indicated by blue ovals. All residue numbers refer to the CYP3A4 sequence (PDB code 1W0E). (DOC) [file pone.0081980.s009.doc]

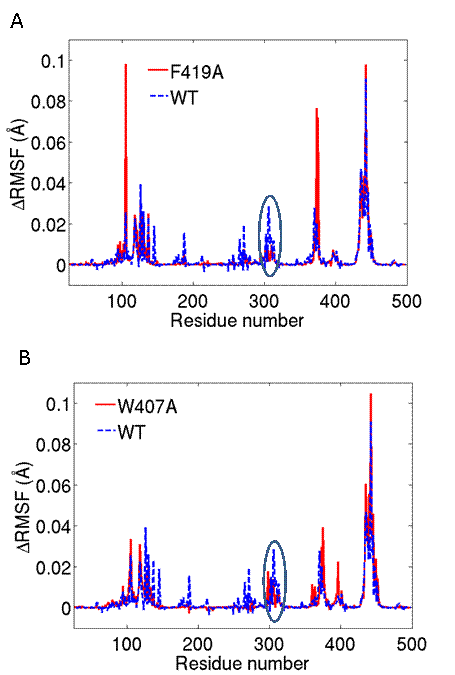


**Figure S9.** The ΔRMSFs of second trajectories for two mutants. (*A)* F419A mutant compared with the WT; (*B*) W407A mutant compared with the WT. The sequence range that encompasses 301-306 is indicated by blue ovals. All residue numbers refer to the CYP3A4 sequence (PDB code 1W0E).
